# Effects of cognitive remediation on negative symptoms dimensions: exploring the role of working memory

**DOI:** 10.1017/S0033291717000757

**Published:** 2017-09-04

**Authors:** M. Cella, D. Stahl, S. Morris, R. S. E. Keefe, M. D. Bell, T. Wykes

**Affiliations:** 1 Department of Psychology, King's College London, Institute of Psychiatry, Psychology & Neuroscience, London, UK; 2 Department of Biostatistics, King's College London, Institute of Psychiatry, Psychology & Neuroscience, London, UK; 3 Division of Adult Translational Research, National Institute of Mental Health, North Bethesda, MD, USA; 4 Department of Psychiatry and Behavioral Sciences, Duke University Medical Center, Durham, NC, USA; 5 Department of Psychiatry, Yale University School of Medicine, New Haven, Connecticut, USA

**Keywords:** Cognition, schizophrenia, negative symptoms, working memory, psychosis

## Abstract

**Background:**

Recent theories suggest that poor working memory (WM) may be the cognitive underpinning of negative symptoms in people with schizophrenia. In this study, we first explore the effect of cognitive remediation (CR) on two clusters of negative symptoms (i.e. expressive and social amotivation), and then assess the relevance of WM gains as a possible mediator of symptom improvement.

**Method:**

Data were accessed for 309 people with schizophrenia from the NIMH Database of Cognitive Training and Remediation Studies and a separate study. Approximately half the participants received CR and the rest were allocated to a control condition. All participants were assessed before and after therapy and at follow-up. Expressive negative symptoms and social amotivation symptoms scores were calculated from the Positive and Negative Syndrome Scale. WM was assessed with digit span and letter-number span tests.

**Results:**

Participants who received CR had a significant improvement in WM scores (*d* = 0.27) compared with those in the control condition. Improvements in social amotivation levels approached statistical significance (*d* = −0.19), but change in expressive negative symptoms did not differ between groups. WM change did not mediate the effect of CR on social amotivation.

**Conclusions:**

The results suggest that a course of CR may benefit behavioural negative symptoms. Despite hypotheses linking memory problems with negative symptoms, the current findings do not support the role of this cognitive domain as a significant mediator. The results indicate that WM improves independently from negative symptoms reduction.

## Introduction

Despite the significance of negative symptoms to the prognosis of schizophrenia treatment options for this symptom cluster are still relatively limited (Messinger *et al.* 2011; Fusar-Poli *et al.* 2015). This may be because we have not defined a target that may impact on these symptoms. One potential mediator has been identified in recent empirical and theoretical work which links negative symptoms and cognitive deficits in people with schizophrenia. In particular, Gold *et al.* (2008) suggest that problems in working memory (WM) may disrupt motivation and the pleasure experience. These authors hypothesised that, in order to recruit motivational resources, individuals use WM to represent events and forecast pleasure. According to this model, deficits in WM could limit the ability to accurately retrieve and use information to motivate and guide future behaviour. This theory has some empirical support as WM performance predicts the accuracy of past pleasure experience (Burbridge & Barch, 2007). More recently, activity in WM brain networks has been associated with improvement in negative symptoms following antipsychotic initiation suggesting that WM may interact with treatment to influence outcomes (Nejad *et al.* 2013). Although a number of hypotheses have been proposed the latter study is the only one to investigate the mediating effect of WM on negative symptoms changes in the context of an intervention.

Although not an elective target for cognitive remediation (CR), the most recent meta-analysis reported that CR has a small but significant effect on symptoms of schizophrenia (Wykes *et al.* 2011). Among studies reporting a positive effect, the majority suggest that the negative symptom cluster is more likely to improve after CR compared with the positive symptom cluster (e.g. Gharaeipour & Scott, 2012; Farreny *et al.* 2013; Sanchez *et al.* 2014; Cella *et al.* 2014*b*). The effect of CR on negative symptoms is consistent with associations between negative symptoms and cognitive problems often found in patients with schizophrenia (Milev *et al.* 2005; Ventura *et al.* 2009). Despite these encouraging results it is still relatively unclear which active ingredients of CR may contribute to negative symptom improvements. The framework proposed by Gold and co-workers (Gold *et al.* 2008; Gold *et al.* 2012) seems a potential candidate to explain how improvements in cognitive domains targeted by CR (e.g. WM) may influence negative symptoms. Indeed, based on this theory Strauss (Strauss, 2013) suggested that CR may be a useful intervention to tackle behavioural negative symptoms.

The current study investigates for the first time the potential benefit of CR for negative symptoms and tests the mediating role of WM as a possible cognitive mechanism. However, the domain of negative symptoms encompasses a wide range of problems, from social behaviour to motivation as well as difficulties in affect display and lack of spontaneity. In an attempt to reduce negative symptom heterogeneity, factor analytic studies have explored solutions that produce more coherent clusters. The overwhelming majority of these studies support two distinct domains: one characterised by expressive deficits, including flat affect and alogia, and the other characterised by behavioural problems such as avolition, asociality and anhedonia (Kirkpatrick and Fischer, 2006; Messinger *et al.* 2011). Distinguishing between these two sub-domains may be important in the context of intervention because the ‘active ingredients’ of therapies may have a selective effect on only one cluster. So in this study we consider these two distinct negative symptom dimensions characterised by: (i) lack of expressivity and (ii) poor social motivation. By exploring mediation pathways this study will test the framework proposed by Gold *et al.* (2008) in the context of an intervention. Change in WM produced by therapy will allow an evaluation of the links between negative symptoms and WM. By analysing the individual level data from different trials, this study will also allow an estimate of how consistent the results will be across different settings and allow a more accurate estimation of the effect size (Riley *et al.* 2010).

This study will not only explore the effects of CR on an understudied target but also investigate the mechanisms responsible for this change by examining potential mediational pathways. CR studies have preferentially focused on evaluating effectiveness, but paid only limited attention to study how effectiveness is achieved (i.e. therapy mechanisms). Work in this direction, even if exploratory, can prove important in improving current intervention approaches and maximise benefits.

In this study, we expect CR to have a selective effect on behavioural negative symptoms and anticipate, in line with Gold *et al.* (2008) that improvement in WM following CR will partly or fully mediate the change in behavioural but not expressive negative symptoms.

## Method

This study used data included in the NIMH Database of Cognitive Training and Remediation Studies (DoCTRS). This database assembled data at the individual level from randomised, controlled trials of CR in people with schizophrenia. For this paper, we analysed data from three studies entered in DoCTRS (Wykes *et al.* 2007; Bell *et al.* 2008; Keefe *et al.* 2012) and data from an unpublished, non-DoCTRS study (Reeder *et al.* Submitted). This latter study is not yet included in the database as it was recently completed and currently under review for publication. These studies were selected because they all assessed WM and negative symptoms.

### Design

In the four trials analysed, CR was compared with a control condition, randomisation was conducted independently and assessors were blind to group allocation. Treatment duration ranged from 12 to 16 weeks. Post-treatment follow-up periods ranged from 24 to 32 weeks. Participants were assessed at intake into the study (Baseline); at the end of treatment (Post-treatment) and at Follow-up. No therapy was provided between post-therapy and follow-up.

### Participants

Participants had a primary diagnosis of schizophrenia or schizoaffective disorder according to DSM-IV criteria and were aged 18–65 years. Exclusion criteria were neurological diseases, traumatic brain injury, a history of learning disability, current substance abuse and poor understanding of English. Participants were recruited in the UK (Wykes *et al.* 2007; Reeder *et al.* Submitted) and in the USA (Bell *et al.* 2007; Keefe *et al.* 2012).

### Therapy

The CR employed consisted primarily of task practice engaging various cognitive domains, including WM, attention, processing speed, long-term memory and executive function. Additional information on the different types of CR used and control conditions is summarised in Table 1.

**Table 1.** Description of the CR interventions considered

	Bell *et al.* (2008)	Keefe *et al.* (2012)	Wykes *et al.* (2007)	Reeder *et al.* (submitted)
*Intervention*	Neurocognitive enhancement therapy + Social information-processing group + Vocational rehabilitation	Posit science brain fitness auditory training + Bridging group	Paper and pencil cognitive remediation tasks	CIRCuiTS is a web-based computerised therapy targeting metacognition
*Intervention length* (*average number of weeks*)	28	8–12	12	12
*Intervention received* (*average*)	113 h	24 sessions	37 sessions	25.5 sessions
*Control condition*^a^	Vocational rehabilitation	Computer games + Healthy lifestyle groups	Treatment as usual	Treatment as usual
*Contact with therapist*	Therapists supported participants for social information processing group	Therapists supported participants for bridging groups	Therapists supported participants throughout the intervention	Therapists supported participants throughout the intervention
*Post-therapy assessment* (*weeks*)	52	12	14	14
*Follow-up assessment* (*in months*)	104	–	40	28

a All active control conditions were matched for contact time.

### Measures

#### Demographic and cognitive data

Basic demographic information was collected on all participants. Premorbid IQ was estimated using the WRAT-R (Kareken *et al.* 1995) or the NAART (Uttl, 2002). Scores were compared using the guidelines proposed by Johnstone *et al.* (1996).

#### Symptoms

Symptoms were assessed with the Positive and Negative Syndrome Scale (PANSS, Kay et al. 1987). This is a 30-item measure of symptom severity for people with schizophrenia. The measure is administered as a clinical interview by a trained researcher or clinician. Each item is scored on a 7-point scale ranging from not symptomatic (i.e. 1) to extremely severe symptom (i.e. 7). For this study, we scored the PANSS according to Liemburg *et al.* (2013) and used the two dimensions of negative symptoms identified in their study: Expressive Negative Symptoms (i.e. flat affect, poor rapport, lack of spontaneity, mannerisms and posturing, motor retardation and avolition) and social amotivation (emotional withdrawal, passive/apathetic social withdrawal and active social avoidance).

#### Working memory

WM was assessed using two well-established tests: the Digit Span test from the Wechsler Adult Intelligence Scale (WAIS–III; Wechsler, 1987) and the Letter-Number Span from the MATRICS Consensus Cognitive Battery (Nuechterlein *et al.* 2008). Only Keefe *et al.* (2012) used the Letter-Number Span. Scores on these tests are reported in standardised *Z*-scores.

### Analysis

The analysis was conducted in two stages. First, we investigated whether individuals randomised to CR showed greater improvement in WM and symptoms compared with TAU at Post-treatment and Follow-up assessments. We conducted a mixed effects analysis based on the intention-to-treat principle. The baseline score of the outcome variable was included as a covariate [analysis of covariance (ANCOVA) approach]. The model considered time (i.e. both Baseline and Follow-up assessments), treatment group and the interaction between time and group as fixed categorical variables. Study was also included as a categorical fixed factor. An unstructured covariance matrix allowing unequal variances and covariances (i.e. correlations) between repeated measures and study was used to account for repeated observations over time (Brown & Prescott, 2006). Maximum-likelihood estimation was used to estimate parameters for models analysing all available data in the presence of missing data, including no observations in Keefe at follow-up (Brown & Prescott, 2006).

Interactions between study and time or treatment, respectively, were assessed using the Bayesian information criteria (BIC). Model selection was performed by assessing change in BIC values with changes larger than 10 providing evidence in support of excluding interaction terms (Raftery, 1995). BIC changes were only reported if the *p* value for the interaction statistics was smaller than 0.1. Cohen's *d* (i.e. predicted mean difference divided by baseline standard deviation) is presented as an estimate of effect size.

Sensitivity analyses were conducted to control for individual studies unduly influencing the results. We reran the models: (i) without Keefe *et al*. to control for missing observations at follow-up, (ii) without each of the other studies in turn to control for potential difference in treatments and (iii) including only participants with completed cases to assess if people with missing data influence the results of the analysis. As WM and negative symptoms vary by age, we conducted a further sensitivity analyses, including age as a covariate in the model. We will report the results of these analyses only when they alter the primary analysis results.

In the second stage, we performed a path analysis to assess possible mediating effects (Baron & Kenny, 1986; MacKinnon & Luecken, 2008). Mediation is a hypothesised causal chain in which one independent variable (i.e. CR) affects a mediating variable (i.e. WM), which, in turn, affects the outcome variable (i.e. social amotivaton or expressive negative symptoms).

Multiple-group path analysis was employed to examine and test whether differences in the path parameters across studies were statistically significant. Testing for cross-group invariance involved comparing a baseline model where all parameters were constrained to be invariant between the groups with a model where no constraints were specified. Path models were fitted using full maximum-likelihood estimation. The Sobel test was used to calculate standard errors of the indirect effects. Comparison of nested models employed a nested Chi-square (χ^2^) test. For all variables we included a path to control for baseline differences in the measures (ANCOVA approach; MacKinnon, 2008). Mixed effects model analyses used STATA 13.1 and path analyses using AMOS 22.

## Results

Data from 309 participants who completed the baseline assessment and were randomised were included in the analyses (i.e. 157 CR and 152 control). Table 1 shows the demographic and clinical characteristics of the two groups at baseline. Positive and general symptoms did not change over time in either groups (Table 2).

**Table 2.** Demographics and clinical characteristics for the two groups

	CR (*N* = 157) Mean (s.d.) or %	Control (*N* = 152) Mean (s.d.)
Gender (male)	68.2%	66.9%
Age (years)	38.3(10.3)	37.1(9.7)
Education (years)	12.8(2.3)	12.4(2.4)
Illness onset over 10 years ago	56.1%	53.7%
Premorbid IQ	93.5(13.1)	91.7(13.6)
PANSS Positive	13.4(5.7)	14(5.9)
PANSS Negative	15.1(6.6)	15.9(6.9)
PANSS General	30.8(9.2)	31.2(9.5)
Medication		
Atypical	68.7%	62.5%
Typical	14.7%	29.2%
Both	16.6%	8.6%

The PANSS factors scores presented are according to Kay *et al*. (1987).

### Does CR directly affect WM?

Participants who received CRT significantly improved their WM compared to those in the control groups at Post-treatment [mean difference: 0.31 (95% CI 0.12–0.50), *z* = 3.16, *p* = 0.002, *d* = 0.27], but the difference was not significant at Follow-up [mean difference: 0.10 (95% CI −0.10 to 0.30), *z* = 1.02, *p* = 0.31, *d* = 0.09]. There was no main effect of study on WM [χ^2^ (3) = 2.74, *p* = 0.43]. The interactions between time and study (*p* = 0.83) and treatment group and study (*p* = 0.62) were not significant and therefore not included in the model. Adding age as a covariate did not influence the outcome (*p* = 0.26).

### Does CR directly affect social amotivation?

The mixed model analyses at post-treatment revealed a trend towards a significant interaction between treatment group and time for social amotivation after controlling for social amotivation baseline [mean difference 0.76 (−0.05 to 1.57) *z* = 1.85, *p* = 0.07, *d* = 0.11]. Participants who received CR showed a significant benefit compared with those in the control groups at post-treatment [mean difference: −1.29 (95% CI −1.79 to −0.78, *z* = −4.99, *p* < 0.0001, *d* = −0.19], but this difference was not maintained at follow-up [mean difference: −0.53 (95% CI −1.34 to 0.29), *z* = −1.27, *p* = 0.21, −0.08]. There were no significant differences between study sites (*p* = 0.27). The interactions between time and study (*p* = 0.63) and treatment group and study (*p* = 0.53) were not significant and not included in the final model. Adding age as a covariate did not influence this result (*p* = 0.6).

### Does CR directly affect expressive negative symptoms?

There were no significant differences in expressive negative symptoms between CR and the control group at Post-treatment [mean difference between CR and TAU = −0.11 (95% CI −0.87 to 0.66), *z* = −0.28, *p* = 0.78, *d* = 0.01] or Follow-up [−0.11 (95% CI −0.97 to 1.05), *z* = 0.08, *p* = 0.94, *d* = 0.01]. There were significant differences between studies [χ^2^(3) = 7.86, *p* = 0.049). The interaction between treatment group and time was not significant [0.15, (95% CI −0.91 to 1.21), *z* = 0.28, *p* = 0.82, *d* = 0.01). The interactions between time and study (*p* = 0.50) and treatment group and study (*p* = 0.06, BIC difference: 11.2) were not significant and not included in the model. Adding age as a covariate did not influence this result (*p* = 0.55) (Table 3).

**Table 3.** Means and standard deviations for expressive negative symptoms, (Exp Neg), social amotivation (Soc Amot) and working memory (WM) for control group and the cognitive remediation (CR) groups at the three assessments points

	Control		CR	*N*
	Mean (s.d.)	*N*	Mean (s.d.)	*N*
Exp Neg				
Baseline	11.47 (5.25)	149	11.70 (5.10)	156
Post-treatment	10.88 (4.66)	137	10.95 (4.54)	146
Follow-up	10.51 (4.83)	93	10.87 (4.17)	93
Soc Amot				
Baseline	6.86 (2.84)	149	7.2 (2.89)	156
Post-treatment	7.19 (3.1)	139	6.11 (2.56)	148
Follow-up	6.5 (3.4)	101	6.18 (2.93)	97
WM				
Baseline	−0.79 (1.21)	147	−0.73 (1.11)	154
Post-treatment	−0.74 (1.24)	136	−0.39 (1.03)	142
Follow-up	−0.67 (1.25)	99	−0.55 (1.01)	101

### Does WM improvement mediate improvements in negative symptoms?

**Fig. 1.**
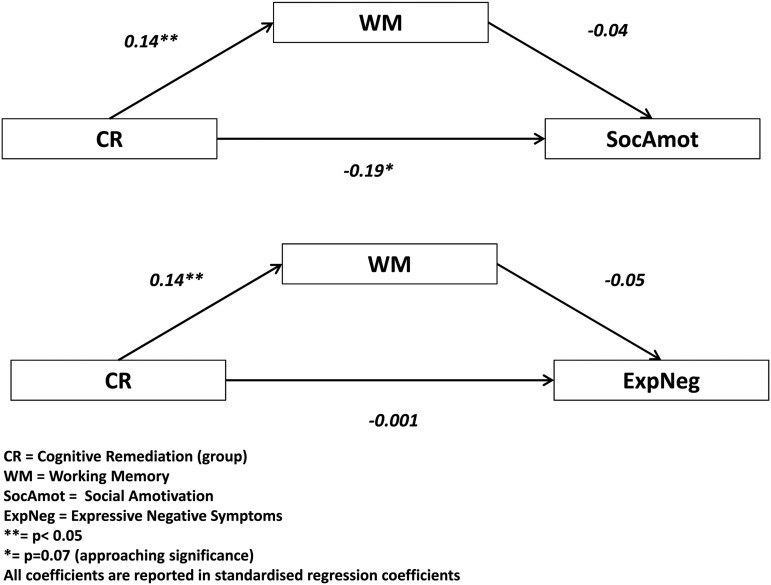
The results of the path analysis investigating the mediating role of WM.

Because the previous analyses revealed no treatment effect in expressive negative symptoms and social amotivation at follow-up we restricted the mediation analysis to the post-therapy time point. A multi-group mediation analysis showed that the CR treatment effect on social amotivation was not mediated by WM [constrained model: indirect effect across groups: *b* = −0.05 (95% CI −0.13 to 0.029), *z* = 1.22, *p* = 0.22, standardised *b*: −0.009] (see Figure 1). The constrained model was significantly different from the unconstrained model [χ^2^(54) = 151.3, *p* < 0.0001] and path models therefore differed among source studies. Standardised indirect effects of individual studies ranged from −0.189 to +0.05 and were all non-significant (Bell: *b* = 0.05, s.e. = 0.10 *z* = 0.44, *p* = 0.66, st.*b* = 0.009, *N* = 77; Wykes:*b* −0.10, s.e. = 0.092, *z* = −1.09, *p* = 0.27, st.b. = −0.018, *N* = 86; Reeder: *b* = −0.189, s.e. = 0.23, *z* = −0.83, *p* = 0.41, st.b. = −0.035, *N* = 93; Keefe: *b* = 0.037, s.e. = 0.09, *z* = 0.63, *p* = 0.53, st.b = 0.00, *N* = 53).

The pooled indirect effect of WM between CR treatment and expressive negative symptoms was close to 0 [constrained model indirect effect: −0.008 (95% CI −0.132 to 0.116), *z* = 1.01, *p* = 0.31, st. beta: −0.001]. Indirect effects of individual studies ranged from −0.155 to 0.027 (standardised betas from −0.017 to 0.003, all non-significant).

## Discussion

Our results further point to a possible beneficial effect of CR on negative symptoms. These analyses, however, show that improvements may only apply to a sub-set of negative symptoms characterised by less expressive and more behavioural features. This effect is evident, at trend significance level, immediately following therapy, but not retained at follow-up. Increasingly, research reports suggest that negative symptom heterogeneity may be a significant factor to consider when evaluating the effects of treatment (Levine & Leucht, 2014). Recent research showed that this is the case in CR for people with schizophrenia (Cella *et al.* 2014*b*) and supports the use of more homogeneous and empirically defined symptoms cluster when evaluating treatment effects. The results of previous studies exploring the effects of CR on the symptoms of schizophrenia might have suffered from conflating information from scales that use heterogeneous symptom clusters with mixed associations with cognitive problems.

Contrary to our hypothesis, the current results suggest that WM does not mediate the effect of CR on social amotivation. This result is at odds with the model proposed suggesting that WM deficits negatively influence reward-related processes and contribute to behavioural negative symptoms (Gold *et al.* 2008). We demonstrated that in the context of an intervention targeting cognitive difficulties, improvement in WM did not contribute to negative symptom change and improvements in these domains occurred independently. This does not rule out the contribution of WM or cognition to negative symptoms. In this study, we could only assess the role of verbal WM on negative symptoms because of the assessment measures used. It is possible, as some studies suggested that visual WM may be contributing more strongly to negative symptoms severity (Pantelis *et al.* 2001). Alternatively, other cognitive domains may be contributing to negative symptoms. Difficulties in planning and organising information may contribute to disorganised behaviour, decreased motivation and less pleasure from experience. Indeed, some research suggests that this may be the case but no specific theory of the mechanism has yet been advanced (Fraguas *et al.* 2014). The set of studies considered in the DoCTRS, database did not allow an investigation of the contribution of executive function because executive function was measured using different tests assessing different competencies that only marginally overlapped (e.g. planning, shifting, inhibition). A planned expansion of the DoCTRS database would allow exploration of this question in the future.

Studies exploring the effects of CR on basic cognitive processes may help us refine our understanding of potential translational mechanism and their relevance to specific symptoms such as the negative symptoms (Cella *et al.* 2015). An example of a promising translational mechanism for negative symptoms is reward sensitivity. Poor sensitivity to feedback has been extensively documented in people with psychosis and is associated with both cognitive difficulties and negative symptom severity (e.g. Gold *et al.* 2008; Strauss *et al.* 2014). A recent study showed that a course of CR is associated with improved sensitivity to feedback and that improvements in this domain are linked to negative symptoms reduction (Cella *et al.* 2014*a*). Future studies should specifically explore the role of reward sensitivity in the context of interventions tackling negative symptoms as this may be a promising mediator.

Alongside improving cognition it is possible that CR may exert a positive effect on negative symptoms via ‘non-specific’ therapy elements including therapeutic alliance and behavioural activation (e.g. session attendance) (e.g. Huddy *et al.* 2012). These aspects are only beginning to be explored and may be particularly important for negative symptoms as they provide social contact opportunities and promote goal-directed behaviour.

There are a limited number of interventions currently available for negative symptoms and these were found to have only a small effect (Fusar-Poli *et al.* 2015). A recent meta-analysis suggested that CR interventions have a moderate effect size on negative symptoms and that this effect is largely durable (Cella *et al.* 2016). The results of this study also suggest that CR programmes using rehabilitation activities alongside cognitive task practice and frequent personal contact with a facilitator or therapist tend to have a higher impact on negative symptoms. It is likely that by providing these elements these programs facilitated learning consolidation and the use of new skills in everyday life. This study demonstrates a similar effect, but in the context of interventions that did not always provide additional rehabilitation activities and intensive therapist contacts. It is possible that by enhancing these two components and focusing task practice on the cognitive domains more strongly associated with negative symptoms a much larger symptom reduction could be observed.

A number of small studies have already attempted to adapt established psychological interventions for psychosis to include a more pronounced focus on negative symptoms; however, the results are not very encouraging (Velthorst *et al.* 2015). The current study used a different approach and took advantage of a database of completed studies to investigate the presence of a ‘signal’. This approach has the benefit of reducing possible bias associated with a therapy delivery method by combining the results of different studies. This approach is efficient as it can be used to test hypotheses without the need to collect new data. Recent evidence suggests that using aggregates of individual data should be preferred, where possible, to traditional meta-analytic studies (Riley *et al.* 2010). Some of the advantages of this methodology include: screening for missing data, including recruitment site in the analysis, replicating results, using standardised analysis across different studies, testing model assumption (e.g. complex interactions between time, treatments and sites) and consistently adjusting for baseline variables. Studies comparing the results of individual data meta-analysis to traditional meta-analytic approaches have shown that differences in the results between these two methods can be sizable and influence practice (e.g. Berlin *et al.* 2002; McCormack *et al.* 2004). This method is preferred as the best source of evidence and should be used more often in mental health research to consolidate current evidence and inform best practice.

This study has limitations. The PANSS, despite assessing negative symptoms, was not designed to capture specific components of this symptom clusters and it may be that the factors used for this study only account for some features of these domains. Although our analysis did not highlight any study behaving as an outlier, it is possible that there may be a group of studies that behave differently and that this trend is not evident due to the restricted numbers of studies. There is a considerable variability in the neuropsychological assessment measures used by different studies and this limits the possibility of aggregating data.

The current analyses also suggest that treatment-related gains in WM and social amotivation may not be maintained at follow-up. This result is at odds with the most recent meta-analysis, which suggests that cognitive gains are durable (Wykes *et al.* 2011). The studies included in our analysis, with the exception of Bell *et al.* (2008), did not provide CR in the context of other comprehensive rehabilitation interventions. There is increasing support for the notion that CR achieves more durable gains when delivered alongside rehabilitation (McGurk *et al.* 2007; Bowie *et al.* 2012). With the majority of the programmes considered here not offering additional support, it is possible that the lack of treatment gain retention may be dependent on lack of opportunities to apply CR gains in a wider rehabilitative context.
